# Dynamics of almost periodic Schoener’s competition model with time delays and impulses

**DOI:** 10.1186/s40064-016-2068-x

**Published:** 2016-04-14

**Authors:** Can Li, Zunguang Guo, Zhiyu Zhang

**Affiliations:** Department of Science, Taiyuan Institute of Technology, Taiyuan, 030008 Shanxi China

**Keywords:** Comparison theorem, Lyapunov functional, Persistence, Almost periodicity, Impulsive differential equations, 34K14, 34K20, 34K45, 92D25

## Abstract

In real world, the ecological systems are usually perturbed by human exploitation activities such as planting and harvesting and so on. In order to obtain a more accurate description for such phenomenon, the impulsive differential equations play an important role. This paper is concerned with a kind of almost periodic Schoener’s competition model with pure-delays and impulsive effects. By using the comparison theorem and the Lyapunov functional method of the impulsive differential equations, some sufficient conditions are obtained for the persistence and existence of a unique uniformly asymptotically stable positive almost periodic solution in a class of impulsive Schoener’s competition model with pure-delays. The main results in this paper improve some results in recent years. And the method used in this paper provides a possible and effective method to study the permanence and existence of a unique uniformly asymptotically stable positive almost periodic solution of the models with impulsive perturbations in biological populations. Finally, an example and numerical simulations are given to illustrate the feasibility and effectiveness of our main results.

## Background

One of the most interest topics in mathematics ecology concerns the uniform persistence, almost periodic oscillations and global stability of ecology system. It is well known that a lot of Lotka–Volterra competitive systems have been discussed. Chen (1988) proposed and discussed a more practical competitive model as follows:

1 $$\begin{aligned} \left\{ \begin{array}{lll} \dot{x}_{1}(t)=x_{1}(t)\bigg [\frac{a_{10}(t)}{x_{1}(t)+m_{1}(t)} -a_{11}(t)x_{1}(t)-a_{12}(t)x_{2}(t)-c_{1}(t)\bigg ],\\ \dot{x}_{2}(t)=x_{2}(t)\bigg [\frac{a_{20}(t)}{x_{2}(t)+m_{2}(t)}-a_{21}(t)x_{1}(t) -a_{22}(t)x_{2}(t)-c_{2}(t)\bigg ]. \end{array}\qquad \right. \end{aligned}$$

In biological terms, $x_1$ and $x_2$ stand for the spatial densities of each species and $c_1$ and $c_2$ are their respective net death rate. The coefficients $a_{11}$ and $a_{22}$ are intra-specific competition rates whereas $a_{12}$ and $a_{21}$ are inter-specific competition rates. The asymptotic behaviors of the solution to the system (1) have been studied in Liu et al. (2006).

It is well known that the assumption of almost periodicity of the coefficients in systems is a way of incorporating the time-dependent variability of the environment, especially when the various components of the environment are periodic with not necessary commensurate periods (e.g., climate change, food supplies, mating habits and harvesting). For this reason, the assumption of almost periodicity is more realistic, more important and more general when we consider the effects of the environmental factors. In recent years, there are many scholars concerning with the Schoener’s competition system. Topics such as existence, uniqueness and global attractivity of positive periodic solutions or almost periodic solutions of the system were extensively investigated, and many excellent results have been derived (see Xue et al. 2015; Tian et al. 2015; Zhang et al. 2015; Liu and Xu 2004; Liu et al. 2006; Li and Yang 2009; Gan and Lin 2012; Wu et al. 2009; Zhang et al. 2012 and the references cited therein).

On the other hand, many evolution processes are characterized by the fact that at certain moments of time they experience a change of state abruptly. These processes are subject to short-term perturbations whose duration is negligible in comparison with the duration of the process. Consequently, it is natural to assume that these perturbations act instantaneously, that is, in the form of impulses. Thus impulsive differential equations, that is, differential equations involving impulse effects, appear as a natural description of observed evolution phenomena of real world problems. The ecological systems are often deeply perturbed by human exploitation activities such as planting and harvesting and so on, which makes them unsuitable to be considered continually. In recent years, the impulsive problems in ecological systems have been intensively investigated (see Lakshmikantham et al. 1989; Stamov 2012; Samoilenko and Perestyuk 1995; Bainov and Simeonov 1993; Jin et al. 2005; Stamov 2009; Liu and Chen 2007; He et al. 2010; Zhang et al. 2014; Zhang and Li 2011 for more detail). For instance, Zhang et al. (2012) studied the following almost periodic Schoener’s competition model with pure-delays and impulsive effects:

2 $$\begin{aligned} \left\{ \begin{array}{lll} \dot{x}_{1}(t)=x_{1}(t)\bigg [\frac{a_{10}(t)}{x_{1}(t-\tau _{10})+m_{1}(t)} -a_{11}(t)x_{1}(t-\tau _{11})-a_{12}(t)x_{2}(t-\tau _{12})-c_{1}(t)\bigg ],\\ \dot{x}_{2}(t)=x_{2}(t)\bigg [\frac{a_{20}(t)}{x_{2}(t-\tau _{20})+m_{2}(t)}-a_{21}(t)x_{1}(t-\tau _{21}) -a_{22}(t)x_{2}(t-\tau _{22})-c_{2}(t)\bigg ],\quad t\ne \tau _k,\\ \Delta x_1(\tau _k)=h_{1k}x_1(\tau _k),\\ \Delta x_2(\tau _k)=h_{2k}x_2(\tau _k),\quad k\in {\mathbb {Z}}^+:=\{0,1,\ldots \}, \end{array} \right. \end{aligned}$$

where $x_{1}(t)$, $x_{2}(t)$ are population densities of species $x_{1}$, $x_{2}$ at time *t*, respectively, $\tau _{ij}$ are all nonnegative integers, $a_{ij}$, $m_{i}$ and $c_{i}$, are all positive almost periodic functions, $i=1,2$, $j=0,1,2$, $h_{1k}, h_{2k}\ge 0$ are almost periodic sequences, $0=\tau _0<\tau _1< \tau _2< \cdots< \tau _k< \tau _{k+1} < \cdots$, are impulse points with $\lim _{k\rightarrow +\infty }\tau _k=+\infty$, and the set of sequences $\{\tau _{k}^{j}\}, \tau _{k}^{j} =\tau _{k+j}-\tau _{k}, k\in {\mathbb {Z}}^+, j\in {\mathbb {Z}}$ is uniformly almost periodic (see Definition 1 in “Preliminaries” section).

The permanence and almost periodicity of all species in multispecies community are very important in biological populations. In recent years, the permanence and almost periodic solution of the models in biological populations has been studied by many authors (see Zhang 2013, 2014; Du and Lv 2013; Meng and Chen 2006; Lin and Chen 2009; Zhou et al. 2013; Zhang et al. 2014; Xia et al. 2008; Xia 2013; Liao and Zhang 2012; Liao and Xu 2014 and the references cited therein). In these cases, the comparison theorems and the Liapunov functional method of differential equations have been applied to obtain the permanence and almost periodic solutions. However, we find few papers referring to the discontinuous systems (i.e., impulsive systems).

Recently, there are some scholars concerning with the permanence and almost periodic solution of the impulsive models in biological populations, see Zhang et al. (2012, 2014), He et al. (2010). For example, He et al. (2010) considered the following impulsive differential equation model of plankton allelopathy

3 $$\begin{aligned} \left\{ \begin{array}{lll} \dot{x}_{1}(t)=x_{1}(t)\bigg [r_1(t) -a_{11}(t)x_{1}(t)-a_{12}(t)x_{1}(t)-b_{1}(t)x_{1}(t)x_2(t)\bigg ],\\ \dot{x}_{2}(t)=x_{2}(t)\bigg [r_1(t) -a_{21}(t)x_{1}(t)-a_{22}(t)x_{2}(t)-b_{2}(t)x_{1}(t)x_2(t)\bigg ],\quad t\ne t_k,\\ \Delta x_1(t_k)=h_{1k}x_1(t_k),\\ \Delta x_2(t_k)=h_{2k}x_2(t_k),\quad k\in {\mathbb {Z}}^+. \end{array} \right. \end{aligned}$$

By using the relation between the solutions of impulsive system and the corresponding non-impulsive system, the authors transformed impulsive system (3) into a continuous system. Further, by means of the comparison theorems and the Liapunov functional method of differential equations without impulses, the authors obtained some sufficient conditions ensuring the existence of a unique uniformly asymptotically stable positive almost periodic solution of system (3).

Next, by using a similar method as that in He et al. (2010), the authors in Zhang et al. (2012, 2014) studied the permanence and almost periodic solution of system (2) and the following impulsive multispecies mutualism system:

4 $$\begin{aligned} \left\{ \begin{array}{ll} \dot{x}_{i}(t)=x_{i}(t)\bigg [r_i(t) -a_{i}(t)x_{i}(t-\tau _i(t))+\sum \nolimits _{j=1,j\ne i}^nb_{ij}(t)\frac{x_j(t)}{1+x_j(t)}\bigg ],\quad t\ne t_{k},\\ \Delta x_i(t_k)=h_{ik}x_i(t_k),\quad k\in {\mathbb {Z}}^+,\quad i=1,2,\ldots ,n. \end{array} \right. \end{aligned}$$

It is worthwhile to note that the main results of paper (Zhang et al. 2012, 2014; He et al. 2010) indicates the impulsive coefficients $h_{ik}$ in system (2)–(4) satisfying the following condition:

(*F*) : $H_{i}(t):=\prod _{0<t_{k}<t}(1+h_{ik})$, $i=1,2$ or $i=1,2,\ldots ,n\,$ (for system (4)), $k\in {\mathbb {Z}}^+$ is almost periodic function and there exist positive constants $H_{i}^{u}$ and $H_{i}^{l}$ such that $H_{i}^{l}\le H_{i}(t)\le H_{i}^{u}$.

### *Remark 1*

Obviously, condition (*F*) is too harsh. For example, if the impulse coefficient $h_{ik}\equiv 0.3\,(i=1,2)$ in system (2)–(4), then (*F*) is invalid. Therefore, the main results in papers (Zhang et al. 2012, 2014; He et al. 2010) are difficult to apply to more extensive model with impulsive effects.

In Zhang et al. (2012, 2014), He et al. (2010), although the authors considered the impulsive system, but still used the research method of continuous systems. Stimulated by this, by means of the comparison theorem and the Lyapunov functional method of the impulsive differential equations (Lakshmikantham et al. 1989; Stamov 2012), the main purpose of this paper is to establish some sufficient conditions which guarantee the permanence and existence of a unique uniformly asymptotically stable positive almost periodic solution of system (2). The main results obtained in this paper remove the harsh condition (*F*) and provide a possible and effective method to study the permanence and existence of a unique uniformly asymptotically stable positive almost periodic solution of the models with impulsive perturbations in biological populations.

Let ${\mathbb {R}}$ and ${\mathbb {Z}}$ denote the sets of real numbers and integers integers, respectively. Related to a continuous function *f*, we use the following notations:

$$f^l=\inf _{s\in {\mathbb {R}}}f(s),\quad f^u=\sup _{s\in {\mathbb {R}}}f(s).$$

The organization of this paper is as follows. In “Preliminaries” section, we give some basic definitions and necessary lemmas which will be used in later sections. In “Permanence” section, by using the comparison theorem of the impulsive differential equations (Lakshmikantham et al. 1989), we give the permanence of system (2). In “Almost periodic solution” section, we study the existence of a unique uniformly asymptotically stable positive almost periodic solution of system (2) by applying the Lyapunov method of the impulsive differential equations (Stamov 2012). Finally, an example and numerical simulations are given to illustrate that our results are feasible.

## Preliminaries

Now, let us state the following definitions and lemmas, which will be useful in proving our main result.

By ${\mathbb {I}}$, ${\mathbb {I}}=\{\{\tau _{k}\}\in {\mathbb {R}}: \tau _{k}<\tau _{k+1}$, $k\in {\mathbb {Z}}$, $\lim _{k\rightarrow \pm \infty }\tau _{k}=\pm \infty \}$, we denote the set of all sequences that are unbounded and strictly increasing. Let $\Omega \subset {\mathbb {R}}$, $\Omega \ne \emptyset$, $\tau =\max \{2\tau _{ij}, i=1,2, j=0,1,2\}$, $\xi _{0}\in {\mathbb {R}}$, introduce the following notations:

$PC(\xi _{0})$ is the space of all functions $\phi :[\xi _{0}-\tau ,\xi _{0}]\rightarrow \Omega$ having points of discontinuity at $\mu _{1},\mu _{2},\ldots \in [\xi _{0}-\tau ,\xi _{0}]$ of the first kind and left continuous at these points.

For $J\subset {\mathbb {R}}$, $PC(J, {\mathbb {R}})$ is the space of all piecewise continuous functions from *J* to ${\mathbb {R}}$ with points of discontinuity of the first kind $\tau _{k}$, at which it is left continuous.

Let $\phi _{1}, \phi _{2}\in PC(0)$. Denote by $x_{i}(t)=x_{i}(t;0,\phi _{i})$, $x_{i}\in \Omega$, $i=1,2$ the solution of system (2) satisfying the initial conditions

5 $$0\le x_{i}(s;0,\phi _{i})=\phi _{i}(s)<+\infty ,\quad s\in [-\tau ,0],\quad x_{i}(0+0;0,\phi _{i})=\phi _{i}(0)>0.$$

By the basic theories of impulsive differential equations with delay in Stamov (2012), system (2) and (5) has a unique solution. Since the solution of system (2) and (5) is a piecewise continuous function with points of discontinuity of the first kind $\tau _{k}$, $k\in {\mathbb {Z}}$ we adopt the following definitions for almost periodicity.

### **Definition 1**

(Stamov 2012) The integer number *p* is said to be an $\epsilon$-almost period of $\{\tau _k\}$, if for $k\in {\mathbb {Z}}$, $|\tau _{k+p}-\tau _k|<\epsilon .$

### **Definition 2**

(Stamov 2012) The set of sequences $\{\tau _{k}^{j}\}, \tau _{k}^{j} =\tau _{k+j}-\tau _{k}, k\in {\mathbb {Z}}, j\in {\mathbb {Z}}, \{\tau _{k}\}\in {\mathbb {I}}$ is said to be uniformly almost periodic if for arbitrary $\epsilon >0$ there exists a relatively dense set of $\epsilon$-almost periods common for any sequences.

### **Definition 3**

(Stamov 2012) The function $\varphi \in PC({\mathbb {R}},{\mathbb {R}})$ is said to be almost periodic, if the following hold:

1. The set of sequences $\{\tau _{k}^{j}\}, \tau _{k}^{j}=\tau _{k+j}- \tau _{k}, k\in {\mathbb {Z}}, j\in {\mathbb {Z}}, \{\tau _{k}\}\in {\mathbb {I}}$ is uniformly almost periodic.
2. For any $\epsilon >0$ there exists a real number $\delta >0$ such that if the points $t'$ and $t''$ belong to one and the same interval of continuity of $\varphi (t)$ and satisfy the inequality $|t'-t''|<\delta$, then $|\varphi (t')-\varphi (t'')|<\epsilon$.
3. For any $\epsilon >0$ there exists a relatively dense set *T* such that if $\eta \in T$ , then $|\varphi (t+\eta )-\varphi (t)|<\epsilon$ for all $t\in {\mathbb {R}}$ satisfying the condition $|t-\tau _{k}|>\epsilon$, $k\in {\mathbb {Z}}$. The elements of *T* are called $\epsilon$-almost periods.

### **Lemma 1**

(Stamov 2012)* Let *$\{\tau _{k}\}\in {\mathbb {I}}$* Then there exists a positive integer*. *A such that on each interval of length 1, we have no more than A elements of the sequence *$\{\tau _{k}\}$*, i.e.,*

$$i(s,t)\le A(t-s)+A,$$

*where i*(*s*, *t*)* is the number of the points*$\tau _{k}$* in the interval *(*s*, *t*).

Theoretically, one can investigate the existence, uniqueness and stability of almost periodic solution for functional differential equations by using Lyapunov functional as follows (Stamov 2012, $\mathrm {P}_{109}$):

Let ${\mathbb {R}}^{n}$ be the *n*-dimensional Euclidean space with elements $x=(x_{1},\ldots ,x_{n})^{T}$ and norm $|x|_{0}=\sum _{i=1}^{n}|x_{i}|$, $C=C([-\tau ,0],{\mathbb {R}}^{n})$, ${\mathbb {B}}\in {\mathbb {R}}^+$. Denote $C_{\mathbb {B}}=\{\varphi \in C:\Vert \varphi \Vert <{\mathbb {B}}\}$, with $\Vert \varphi \Vert =\sup _{s\in [-\tau ,0]}|\varphi (s)|_{0}$.

Consider the system of impulsive differential equations with delay:

6 $$\begin{aligned} \left\{ \begin{array}{lll} \dot{x}(t)=f(t,x_t),\quad t\ne \tau _k,\\ \Delta x(\tau _k)=I_{k}(x(\tau _k)), \end{array} \right. \end{aligned}$$

where $t\in {\mathbb {R}}$, $\{\tau _k\}\in {\mathbb {I}}$, $f(t,\varphi )$ is continuous in $(t,\varphi )\in {\mathbb {R}}\times C_{\mathbb {B}}$ and almost periodic in *t* uniformly for $\varphi \in C_{\mathbb {B}}$, $\forall \rho >0$, $\exists M(\rho )>0$ such that $|f(t,\varphi )|\le M(\rho )$ as $t\in {\mathbb {R}}$, $\varphi \in C_{\mathbb {\rho }}$, while $x_{t}\in C_{\mathbb {B}}$ is defined as $x_{t}(s)=x(t+s)$ for $s\in [-\tau ,0]$, $I_k: D\rightarrow {\mathbb {R}}^n$, $k\in {\mathbb {Z}}$, *D* is an open set in ${\mathbb {R}}^n$.

Introduce the following conditions:

$$(C_1)$$
The sequence $\{I_k(x)\}$, $k\in {\mathbb {Z}}$ is almost periodic uniformly with respect to $x\in D$.

### **Lemma 2**

(Stamov 2012, ${\mathrm {P}}_{109}$)* Suppose that there exists a Lyapunov functional *$V(t,\phi ,\psi )$* defined on*${\mathbb {R}}\times C_{{\mathbb {B}}}\times C_{\mathbb {B}}$* satisfying the following conditions:*

1. $u(\Vert \phi -\psi \Vert )\le V(t,\phi ,\psi )\le v(\Vert \phi -\psi \Vert )$,* where*$u, v\in {\mathcal {P}}$* with*${\mathcal {P}}=\{u:{\mathbb {R}}^{+}\rightarrow {\mathbb {R}}^{+}|u$* is continuous increasing function and*$u(s)\rightarrow 0$* as*$s\rightarrow 0\}$.
2. $|V(t,\bar{\phi },\bar{\psi })-V(t,\hat{\phi },\hat{\psi })|\le {\mathrm {L}}(\Vert \bar{\phi }-\hat{\phi }\Vert +\Vert \bar{\psi }-\hat{\psi }\Vert )$,* where*${\mathrm {L}}>0$* is a constant.*
3. *For*$t=\tau _k$, $V(t^+,\phi +I_k(\phi ),\psi +I_k(\psi ))\le V(t,\phi ,\psi )$;* For*$t\ne \tau _k$, $\dot{V}_{(2.2)}(t,\phi ,\psi )\le -\gamma V(t,\phi ,\psi )$, $\forall k\in {\mathbb {Z}}$,* where*$\gamma >0$* is a constant*.

Moreover, one assumes that system (6) has a solution that remains in a compact set $S\subset D$. Then system (6) has a unique almost periodic solution which is uniformly asymptotically stable.

### *Remark 2*

From the proof of Lemma 2, it is not difficult to prove that condition **(1)** of Lemma 2 can be replaced by the following condition:

1. $u(|\phi (0)-\psi (0)|_{0})\le V(t,\phi ,\psi )\le v(\Vert \phi -\psi \Vert )$, where $u, v\in {\mathcal {P}}$ with ${\mathcal {P}}=\{u:{\mathbb {R}}^{+}\rightarrow {\mathbb {R}}^{+}|u$ is continuous increasing function and $u(s)\rightarrow 0$ as $s\rightarrow 0\}$.

## Permanence

In this section, we establish a permanence result for system (2).

### **Lemma 3**

(Lakshmikantham et al. 1989)* Assume that*$x\in PC({\mathbb {R}})$* with points of discontinuity at*$t=\tau _k$* and is left continuous at*$t=\tau _k$* for*$k\in {\mathbb {Z}}^+$,* and*

7 $$\left\{ \begin{array}{lll} \dot{x}(t)\le f(t,x(t)), &\quad t\ne \tau _k,\\ x(\tau _k^+)\le I_{k}(x(\tau _k)), &\quad k\in {\mathbb {Z}}^+, \end{array} \right.$$

*where*$f\in C({\mathbb {R}}\times {\mathbb {R}},{\mathbb {R}})$, $I_k\in C({\mathbb {R}},{\mathbb {R}})$* and*$I_k(x)$* is nondecreasing inx for*$k\in {\mathbb {Z}}^+$.* Let*$u^*(t)$* be the maximal solution of the scalar impulsive differential equation*

8 $$\left\{ \begin{array}{lll} \dot{u}(t)=f(t,u(t)),&\quad t\ne \tau _k,\\ u(\tau _k^+)=I_{k}(u(\tau _k))\ge 0, &\quad k\in {\mathbb {Z}}^+,\\ u(t_0^+)=u_0 \end{array} \right.$$

*existing on*$[t_0,\infty )$.* Then*$x(t_0^+)\le u_0$* implies*$x(t)\le u^*(t)$* for*$t\ge t_0$.

### *Remark 3*

If the inequalities (7) in Lemma 3 is reversed and $u_*(t)$ is the minimal solution of system (8) existing on $[t_0,\infty )$, then $x(t_0^+)\ge u_0$ implies $x(t)\ge u_*(t)$ for $t\ge t_0$.

For arbitrary $a, b>0$, $h_k\ge 0$, we give the following notations:

$$\begin{aligned} \xi: &= \ln \sup _{k\in {\mathbb {Z}}}\frac{1}{1+h_k},\quad \alpha :=a-\xi A,\quad \theta :=\inf _{k\in {\mathbb {Z}}}\tau _k^1,\quad \eta :=\inf _{k\in {\mathbb {Z}}}\bigg \{\prod _{j=0}^1\frac{1}{1+h_{j+k}},1\bigg \},\\ W(t,s) &=\left\{ \begin{array}{ll} e^{-a(t-s)}, &{}\tau _{k-1}<s<t<\tau _{k}; \\ \prod \nolimits _{j=m}^{k+1}\frac{1}{1+h_j} e^{-a(t-s)}, \qquad &{}\tau _{m-1}<s\le \tau _{m}<\tau _{k}<t\le \tau _{k+1}, \end{array} \right. \end{aligned}$$

where *A* is defined as that in Lemma 1, $\tau _k^1=\tau _{k+1}-\tau _k$ is defined as that in Definition 2.

### **Lemma 4**

*Assume that*$a, b>0$, $h_k\ge 0$,* then the following impulsive logistic equation*

9 $$\begin{aligned} \left\{ \begin{array}{lll} \dot{x}(t)=x(t)\big [ a-bx(t)\big ],\quad t\ne \tau _k,\\ \Delta x(\tau _k)=h_{k}x(\tau _k),\quad k\in {\mathbb {Z}}^+ \end{array} \right. \end{aligned}$$

*has a unique globally asymptotically stable positive almost periodic solution *$x^*$*, which can be expressed as follows:*

10 $$\frac{\alpha }{e^{\xi A}b}\le x^*(t)=\bigg [b\int _{-\infty }^{t}W(t,s)\,{\mathrm {d}}s\bigg ]^{-1}\le \frac{a}{\eta b(1-e^{-a\theta })}.$$

### *Proof*

Let $u=\frac{1}{x}$, then system (9) changes to

11 $$\left\{ \begin{array}{lll} \frac{{\mathrm {d}}u(t)}{{\mathrm {d}}t}= -au(t)+b,&\quad t\ne \tau _k,\\ \Delta u(\tau _k)=-\frac{h_k}{1+h_{k}}u(\tau _k),&\quad k\in {\mathbb {Z}}^+. \end{array} \right.$$

Together with the system (11) we consider the linear system

12 $$\left\{ \begin{array}{lll} \frac{{\mathrm {d}}u(t)}{{\mathrm {d}}t}= -au(t),&\quad t\ne \tau _k,\\ \Delta u(\tau _k)=-\frac{h_k}{1+h_{k}}u(\tau _k),&\quad k\in {\mathbb {Z}}^+. \end{array} \right.$$

Now let us consider the equation

$$\frac{{\mathrm {d}}u(t)}{{\mathrm {d}}t}= -au(t),\quad \tau _{k-1}< t\le \tau _{k}$$

and its solution

$$u(t)=u(s)e^{-a(t-s)},\quad \tau _{k-1}<s<t\le \tau _{k}.$$

Then from Stamov (2012), the Cauchy matrix of the linear system (12) is

$$W(t,s)=\left\{ \begin{array}{ll} e^{-a(t-s)}, &{}\tau _{k-1}<s<t<\tau _{k}; \\ \prod \nolimits _{j=m}^{k+1}\frac{1}{1+h_j} e^{-a(t-s)}, \quad &{}\tau _{m-1}<s\le \tau _{m}<\tau _{k}<t\le \tau _{k+1} \end{array} \right.$$

and the solution of system (12) is in the form

$$u(t;t_{0};u(t_{0}))=W(t,t_{0}) u(t_{0}),\quad t_{0}\in {\mathbb {R}}.$$

Therefore, system (11) has a solution

$$u(t;t_{0};u(t_{0}))=W(t,t_0)u(t_0)+b\int _{t_0}^{t}W(t,s)\,{\mathrm {d}}s.$$

Letting $t_0\rightarrow -\infty$ in the above equation ($W(t,t_0)u(t_0)\rightarrow 0$), then by Stamov (2012) we have

$$u(t)=b\int _{-\infty }^{t}W(t,s)\,{\mathrm {d}}s$$

is a solution of system (11) and is almost periodic. Then system (9) has a almost periodic solution $x^*(t)$ which can be expressed by (10). By Lemma 1, we have from (10) that

$$\begin{aligned} x^*(t) \ge \bigg [b\int _{-\infty }^{t}e^{i(s,t)\xi }e^{-a(t-s)}\,{\mathrm {d}}s\bigg ]^{-1} \ge \bigg [b\int _{-\infty }^{t}e^{\xi A}e^{-\alpha (t-s)}\,{\mathrm {d}}s\bigg ]^{-1} =\frac{\alpha }{e^{\xi A}b}. \end{aligned}$$

On the other hand,

$$\begin{aligned} x^*(t) \le \bigg [b\int _{t-\theta }^{t}W(t,s)\,{\mathrm {d}}s\bigg ]^{-1} \le \bigg [b\int _{t-\theta }^{t}\eta e^{-a(t-s)}\,{\mathrm {d}}s\bigg ]^{-1} =\frac{a}{\eta b(1-e^{-a\theta })}. \end{aligned}$$

Next, we shall prove that the uniqueness and stability of $x^*(t)$ of system (9). Suppose that *x*(*t*) is another positive solution of system (9). Define a function

$$V(t)=|\ln x^*(t)-\ln x(t)|, \quad \forall t\in {\mathbb {R}}.$$

For $t\ne \tau _k$, $k\in {\mathbb {Z}}^+$, calculating the upper right derivative of *V*(*t*) along the solution of system (9), we have

13 $$D^+V(t)={\mathrm {sgn}}[x^*(t)-x(t)]\bigg [\frac{\dot{x}^*(t)}{x^*(t)}-\frac{\dot{x}(t)}{x(t)}\bigg ] =-b|x^*(t)-x(t)|.$$

For $t=\tau _k$, $k\in {\mathbb {Z}}^+$, we have

$$\begin{aligned} V(\tau _k^+) &= |\ln x^*(\tau _k^+)-\ln x(\tau _k^+)|\\&= \left| \ln \frac{(1+h_{k})x^*(\tau _k)}{(1+h_{k})x(\tau _k)}\right| =|\ln x^*(\tau _k)-\ln x(\tau _k)|=V(\tau _k). \end{aligned}$$

Therefore, *V* is non-increasing. Integrating (13) from 0 to *t* leads to

$$V(t)+b\int _{0}^t|x(s)-x^*(s)|\,{\mathrm {d}}s \le V(0)<+\infty , \quad \forall t\ge 0,$$

that is,

$$\int _{0}^{+\infty }|x(s)-x^*(s)|\,{\mathrm {d}}s<+\infty ,$$

which implies that

$$\lim _{s\rightarrow +\infty }|x(s)-x^*(s)|=0.$$

Thus, the almost periodic solution of system (9) is globally asymptotically stable. This completes the proof.

### **Lemma 5**

*Assume that*$a, b>0$, $h_k\ge 0$,* then every solutionx of the following system with delay*

14 $$\left\{ \begin{array}{lll} \dot{x}(t)\le x(t)\big [ a-bx(t-\tau )\big ],\quad t\ne \tau _k,\\ \Delta x(\tau _k)= h_{k}x(\tau _k),\quad k\in {\mathbb {Z}}^+ \end{array} \right.$$

*satisfies*

$$\lim \sup _{t\rightarrow \infty }x(t)\le M:=\frac{a}{\eta B(1-e^{-a\theta })},$$

*where*$$B=\inf _{t\in {\mathbb {R}}}b\prod _{\tau _k\in [t-\tau ,t)}(1+h_k)^{-1}e^{-a\tau }$$, $$\theta :=\inf \nolimits_{k\in {\mathbb {Z}}}\tau_k^1$$* and*$$\eta :=\inf \nolimits _{k\in {\mathbb {Z}}}\prod _{j=1}^2\frac{1}{1+h_{j+k}}$$.

### *Proof*

From system (14), we have

$$\begin{aligned} \left\{ \begin{array}{lll} \dot{x}(t)\le ax(t),\quad t\ne \tau _k,\\ \Delta x(\tau _k)=h_{k}x(\tau _k),\quad k\in {\mathbb {Z}}^+ \end{array} \right. \end{aligned}$$

is equivalent to

15 $$\begin{aligned} \left\{ \begin{array}{lll} \frac{{\mathrm {d}}}{{\mathrm {dt}}}[x(t)e^{-at}]\le 0,\quad t\ne \tau _k,\\ \Delta x(\tau _k)=h_{k}x(\tau _k),\quad k\in {\mathbb {Z}}^+. \end{array} \right. \end{aligned}$$

For some $t\in [0,+\infty )$ and $t\ne \tau _k$, $k\in {\mathbb {Z}}^+$, consider interval $[t-\tau ,t)$. Assume that $\tau _1<\tau _2<\cdots <\tau _j$ are the impulse points in $[t-\tau ,t)$. Integrating the first inequality of system (15) from $t-\tau$ to $\tau _1$ leads to

$$x(\tau _1)e^{-a\tau _1}\le x(t-\tau )e^{-a(t-\tau )}.$$

Integrating the first inequality of system (15) from $\tau _{1}$ to $\tau _2$ leads to

$$x(\tau _{2})e^{-a\tau _{2}}\le x(\tau _{1}^+)e^{-a\tau _{1}}=(1+h_{1})x(\tau _{1})e^{-a\tau _{1}} \le (1+h_{1})x(t-\tau )e^{-a(t-\tau )}.$$

Integrating the first inequality of system (15) from $\tau _{2}$ to $\tau _3$ leads to

$$x(\tau _{3})e^{-a\tau _{3}}\le x(\tau _{2}^+)e^{-a\tau _{2}}=(1+h_{2})x(\tau _{2})e^{-a\tau _{2}} \le (1+h_{1})(1+h_{2})x(t-\tau )e^{-a(t-\tau )}.$$

Repeating the above process, integrating the first inequality of system (15) from $\tau _j$ to *t* leads to

$$x(t)e^{-at}\le x(\tau _j^+)e^{-a\tau _j}=(1+h_j)x(\tau _j)e^{-a\tau _j}\le \prod _{\tau _k\in [t-\tau ,t)}(1+h_k)x(t-\tau )e^{-a(t-\tau )}.$$

Then

16 $$x(t-\tau )\ge \prod _{\tau _k\in [t-\tau ,t)}(1+h_k)^{-1}e^{-a\tau }x(t).$$

Substituting (16) into system (14) leads to

$$\begin{aligned} \left\{ \begin{array}{lll} \dot{x}(t)\le x(t)\big [ a-Bx(t)\big ],\quad t\ne \tau _k,\\ \Delta x(\tau _k)=h_{k}x(\tau _k),\quad k\in {\mathbb {Z}}^+. \end{array} \right. \end{aligned}$$

Consider the auxiliary system

17 $$\begin{aligned} \left\{ \begin{array}{lll} \dot{z}(t)=z(t)\big [ a-Bz(t) \big ],\quad t\ne \tau _k,\\ z(\tau _k^+)=(1+h_{k})z(\tau _k),\quad k\in {\mathbb {Z}}^+. \end{array} \right. \end{aligned}$$

By Lemma 3, $x(t)\le z(t)$, where *z*(*t*) is the solution of system (17) with $z(0^+)=x(0^+)$. By Lemma 4, system (17) has a unique globally asymptotically stable positive almost periodic solution $z^*$ which can be expressed as follows:

$$\begin{aligned} z^*(t)=\bigg [B\int _{-\infty }^{t}W(t,s)\,{\mathrm {d}}s\bigg ]^{-1} \le \bigg [B\int _{t-\theta }^{t}W(t,s)\,{\mathrm {d}}s\bigg ]^{-1} \le \frac{a}{\eta B(1-e^{-a\theta })}:=M. \end{aligned}$$

Then for any constant $\epsilon >0$, there exists $T_1>0$ such that $x(t)\le z(t)<z^*(t)+\epsilon \le M+\epsilon$ for $t>T_1$. So

$$\lim \sup _{t\rightarrow \infty }x(t)\le M.$$

This completes the proof. $\square$

### **Lemma 6**

*Assume that*$a, b>0$, $h_k\ge 0$,* then every solutionx of the following system with delay*

18 $$\begin{aligned} \left\{ \begin{array}{lll} \dot{x}(t)\ge x(t)\big [ a-bx(t-\tau )\big ],\quad t\ne \tau _k,\\ \Delta x(\tau _k)=h_{k}x(\tau _k),\quad k\in {\mathbb {Z}}^+ \end{array} \right. \end{aligned}$$

*satisfies*

$$\lim \inf _{t\rightarrow \infty }x(t)\ge N:=\frac{a-\xi A}{e^{\xi A}D},$$

*where*$D=\sup _{t\in {\mathbb {R}}}b\prod _{\tau _k\in [t-\tau ,t)}(1+h_k)^{-1}e^{-(a-bM)\tau }$, $\xi :=\ln \sup \nolimits _{k\in {\mathbb {Z}}}\frac{1}{1+h_k}$*and A is defined as that in Lemma 1*.

### *Proof*

According to Lemma 5, there exist $\epsilon >0$ and $T_2>0$ such that

$$x(t)\le M+\epsilon \quad \text { for } t\ge T_{2}.$$

From system (18), we have

$$\begin{aligned} \left\{ \begin{array}{lll} \dot{x}(t)\ge [a-b(M+\epsilon )]x(t),\quad t\ne \tau _k,\quad t\ge T_{2},\\ \Delta x(\tau _k)=h_{k}x(\tau _k),\quad k\in {\mathbb {Z}}^+ \end{array} \right. \end{aligned}$$

is equivalent to

$$\begin{aligned} \left\{ \begin{array}{lll} \frac{{\mathrm {d}}}{\mathrm {dt}}[x(t)e^{-[a-b(M+\epsilon )]t}]\ge 0,\quad t\ne \tau _k\quad t\ge T_{2},\\ \Delta x(\tau _k)=h_{k}x(\tau _k),\quad k\in {\mathbb {Z}}^+. \end{array} \right. \end{aligned}$$

Similar to the argument as that in (16), we have

19 $$bx(t-\tau )\le b\prod _{\tau _k\in [t-\tau ,t)}(1+h_k)^{-1}e^{-[a-b(M+\epsilon )]\tau }x(t):=D_\epsilon x(t),\quad t\ge T_{2}.$$

Substituting (19) into system (18) leads to

$$\begin{aligned} \left\{ \begin{array}{lll} \dot{x}(t)\ge x(t)\big [ a-D_\epsilon x(t)\big ],\quad t\ne \tau _k,\quad t\ge T_{2},\\ \Delta x(\tau _k)=h_{k}x(\tau _k),\quad k\in {\mathbb {Z}}^+. \end{array} \right. \end{aligned}$$

Consider the auxiliary system

20 $$\begin{aligned} \left\{ \begin{array}{lll} \dot{z}(t)=z(t)\big [ a-D_\epsilon z(t) \big ],\quad t\ne \tau _k,\quad t\ge T_{2},\\ z(\tau _k^+)=(1+h_{k})z(\tau _k),\quad k\in {\mathbb {Z}}^+. \end{array} \right. \end{aligned}$$

By Remark 3, $x(t)\ge z(t)$, where *z*(*t*) is the solution of system (20) with $z(0^+)=x(0^+)$. By Lemma 4, system (20) has a unique globally asymptotically stable positive almost periodic solution $z^*$ which can be expressed as follows:

$$\begin{aligned} z^*(t)=\bigg [D_\epsilon \int _{-\infty }^{t}W(t,s)\,{\mathrm {d}}s\bigg ]^{-1} \ge \bigg [D_\epsilon \int _{t-\theta }^{t}W(t,s)\,{\mathrm {d}}s\bigg ]^{-1} \ge \frac{a-\xi A}{e^{\xi A}D_\epsilon }. \end{aligned}$$

Letting $\epsilon \rightarrow 0$ in the above inequality leads to

$$z^*(t) \ge \frac{a-\xi A}{e^{\xi A}D}:=N.$$

Similar to the argument as that in Lemma 5, it follows that

$$\lim \inf _{t\rightarrow \infty }x(t)\ge N.$$

This completes the proof. $\square$

### *Remark 4*

When $h_{ik}(i=1,2)\equiv 0$ in systems (14) and (18), then Lemmas 5, 6 change to the corresponding results in Nakata and Muroya (2010). So Lemmas  5, 6 extend the corresponding result in Nakata and Muroya (2010).

Let

$$\eta _i:=\inf _{k\in {\mathbb {Z}}}\prod _{j=0}^1\frac{1}{1+h_{i(j+k)}},\quad \xi _i:=\ln \sup _{k\in {\mathbb {Z}}}\frac{1}{1+h_{ik}},\quad i=1,2.$$

### **Proposition 1**

*Every solution*$(x_1,x_2)^T$* of system* (2)* satisfies*

$$\lim \sup \limits _{t\rightarrow \infty }x_i(t)\le M_i,\quad i=1,2,$$

*where*$M_1$* and*$M_2$* are defined as that in* (21)* and* (22),* respectively*.

### *Proof*

From system (1), we have

$$\begin{aligned} \left\{ \begin{array}{lll} \dot{x}_{1}(t)\le x_{1}(t)\big [r_1^u -a_{11}^lx_{1}(t-\tau _{11})\big ],\quad t\ne \tau _k,\\ x_1(\tau _k^+)=(1+h_{1k})x_1(\tau _k),\quad k\in {\mathbb {Z}}^+, \end{array} \right. \end{aligned}$$

where $r_1^u:=\sup _{t\in {\mathbb {R}}}\big |\frac{a_{10}(t)}{m_{1}(t)}-c_{1}(t)\big |$. By Lemma  5, we have

21 $$\lim \sup _{t\rightarrow \infty }x_1(t)\le M_1:=\frac{r_1^u}{\eta _1 B_1(1-e^{-r_1^u\theta })},$$

where $B_1=\inf _{t\in {\mathbb {R}}}a_{11}^l\prod _{\tau _k\in [t-\tau _{11},t)}(1+h_{1k})^{-1}e^{-r_1^u\tau _{11}}$. Similarly, ones obtain that

22 $$\lim \sup _{t\rightarrow \infty }x_2(t)\le M_2:=\frac{r_2^u}{\eta _2 B_2(1-e^{-r_2^u\theta })},$$

where $r_2^u:=\sup _{t\in {\mathbb {R}}}\big |\frac{a_{20}(t)}{m_{2}(t)}-c_{2}(t)\big |$, $B_2=\inf _{t\in {\mathbb {R}}}a_{22}^l\prod _{\tau _k\in [t-\tau _{22},t)}(1+h_{2k})^{-1}e^{-r_2^u\tau _{22}}$. This completes the proof. $\square$

### **Proposition 2**

*Let*$N_1$* and*$N_2$* are defined as that in* (23)* and* (24),* respectively. Then every solution*$(x_1,x_2)^T$* of system* (2)* satisfies*

$$\lim \inf \limits _{t\rightarrow \infty }x_i(t)\ge N_i,\quad i=1,2,$$

*if the following condition holds:*

$$(H_{1})$$
$$r_1^l:=\inf _{t\in {\mathbb {R}}}\big [\frac{a_{10}(t)}{M_1+m_{1}(t)} -a_{12}(t)M_2-c_{1}(t)\big ]\ge \xi _1A$$, $$r_2^l:=\inf _{t\in {\mathbb {R}}}\big [\frac{a_{20}(t)}{M_2+m_{2}(t)} -a_{21}(t)M_1-c_{2}(t)\big ]\ge \xi _2A.$$

### *Proof*

According to Proposition 1 and $(H_1)$, there exist $\epsilon >0$ and $T_3>0$ such that

$$\begin{aligned}&x_i(t)\le M_i+\epsilon \quad \text { for } t\ge T_{3},\quad i=1,2,\\&r_1^l(\epsilon ):=\inf _{t\in {\mathbb {R}}}\bigg [\frac{a_{10}(t)}{(M_1+\epsilon )+m_{1}(t)} -a_{12}(t)(M_2+\epsilon )-c_{1}(t)\bigg ]\ge \xi _1A,\\&r_2^l(\epsilon ):=\inf _{t\in {\mathbb {R}}}\bigg [\frac{a_{20}(t)}{(M_2+\epsilon )+m_{2}(t)} -a_{21}(t)(M_1+\epsilon )-c_{2}(t)\bigg ]\ge \xi _2A. \end{aligned}$$

From system (1), we have

$$\begin{aligned} \left\{ \begin{array}{lll} \dot{x}_{1}(t)\ge x_{1}(t)\big [r_{1}^l(\epsilon ) -a_{11}^ux_{1}(t-\tau _{11})\big ],\quad t\ne \tau _k, \; t>T_3,\\ x_1(\tau _k^+)=(1+h_{1k})x_1(\tau _k),\quad k\in {\mathbb {Z}}^+. \end{array} \right. \end{aligned}$$

By Lemma 6 and the arbitrariness of $\epsilon$, one has

23 $$\lim \inf _{t\rightarrow \infty }x_1(t)\ge N_1:=\frac{r_{1}^l-\xi _1 A}{e^{\xi _1 A}D_1},$$

where $D_1:=\sup _{t\in {\mathbb {R}}}a_{11}^u\prod _{\tau _k\in [t-\tau _{11},t)}(1+h_{1k})^{-1}e^{-(r_1^l-a_{11}^uM_1)\tau _{11}}$. Similarly, we have

24 $$\lim \inf _{t\rightarrow \infty }x_2(t)\ge N_2:=\frac{r_{2}^l-\xi _2 A}{e^{\xi _2 A}D_2},$$

where $D_2:=\sup _{t\in {\mathbb {R}}}a_{22}^u\prod _{\tau _k\in [t-\tau _{22},t)}(1+h_{2k})^{-1}e^{-(r_2^l-a_{22}^uM_2)\tau _{22}}$. This completes the proof. $\square$

### *Remark 5*

In view of $(H_1)$ in Proposition 2, the values of impulse coefficients $h_{ik}\,(i=1,2)$ and the number of the impulse points $\tau _k$ in each interval of length 1 have negative effect on the permanence of system (2).

By Propositions 1, 2, we have

### **Theorem 1**

*Assume that*$(H_1)$* holds, then system* (2)* is permanent*.

### *Remark 6*

Theorem 1 is a permanence result of system (2) without (*F*). So Theorem 1 improves the corresponding result in Zhang et al. (2012). Further, Theorem 1 provides a possible and effective method to study the permanence of the models with impulsive perturbations and pure-delays in biological populations.

### *Remark 7*

From the proof of Propositions 1, 2, we know that under the conditions of Theorem 1, the set $S=\{(x_1,x_2)^T\in {\mathbb {R}}^2:N_i\le x_i\le M_i, i=1,2\}$ is an invariant set of system (2).

## Almost periodic solution

The main result of this paper concerns the existence of a unique uniformly asymptotically stable positive almost periodic solution for system (2).

For convenience, we introduce some notations as follows:

$$\begin{aligned} \alpha _{1} &= {} \frac{a_{10}^{l}N_{1}}{\big (M_{1}+m_{1}^{u}\big )^{2}}+a_{11}^{l}N_{1} -\bigg (\frac{\sqrt{\tau _{10}}a_{10}^{u}M_{1}}{\big (N_{1}+m_{1}^{l}\big )^{2}}\bigg )^{2} -\frac{(\tau _{10}+\tau _{11})a_{10}^{u}a_{11}^{u}M_{1}^{2}}{\big (N_{1}+m_{1}^{l}\big )^{2}} -\tau _{11}a_{11}^{u2}M_{1}^{2},\\ \alpha _{2} &= \frac{a_{20}^{l}N_{2}}{\big (M_{2}+m_{2}^{u}\big )^{2}}+a_{22}^{l}N_{2} -\bigg [\frac{\sqrt{\tau _{20}}a_{20}^{u}M_{2}}{\big (N_{2}+m_{2}^{l}\big )^{2}}\bigg ]^{2} -\frac{(\tau _{20}+\tau _{22})a_{20}^{u}a_{22}^{u}M_{2}^{2}}{\big (N_{2}+m_{2}^{l}\big )^{2}} -\tau _{22}a_{22}^{u2}M_{2}^{2},\\ \beta _{1} &= \frac{\tau _{20}a_{20}^{u}a_{21}^{u}M_{1}M_{2}}{\big (N_{2}+m_{2}^{l}\big )^{2}}+\tau _{22}a_{21}^{u}a_{22}^{u}M_{1}M_{2} +a_{21}^{u}M_{1},\\ \beta _{2} &= \frac{\tau _{10}a_{10}^{u}a_{12}^{u}M_{1}M_{2}}{\big (N_{1}+m_{1}^{l}\big )^{2}} +\tau _{11}a_{11}^{u}a_{12}^{u}M_{1}M_{2}+a_{12}^{u}M_{2}, \end{aligned}$$

where $M_1$, $M_2$, $N_1$ and $N_2$ are defined as that in “Permanence” section.

### **Theorem 2**

*Assume that*$(H_1)$* holds. Suppose further that *

$$(H_2)$$
*There exist two positive constants*$\lambda _{1}$* and*$\lambda _{2}$* such that*$\lambda _{1}\alpha _{1}>\lambda _{2}\beta _{1}$* and*$\lambda _{2}\alpha _{2}>\lambda _{1}\beta _{2}$.

* Then system* (2)* admits a unique uniformly asymptotically stable almost periodic solution.*

### *Proof*

Let $x_{i}(t)=e^{z_{i}(t)}$, $i=1, 2$, then system (2) is transformed into

25 $$\begin{aligned} \left\{ \begin{array}{lll} \dot{z}_{1}(t)=\frac{a_{10}(t)}{e^{z_{1}(t-\tau _{10})}+m_{1}(t)} -a_{11}(t)e^{z_{1}(t-\tau _{11})}-a_{12}(t)e^{z_{2}(t-\tau _{12})}-c_{1}(t),\\ \dot{z}_{2}(t)=\frac{a_{20}(t)}{e^{z_{2}(t-\tau _{20})}+m_{2}(t)}-a_{21}(t)e^{z_{1}(t-\tau _{21})} -a_{22}(t)e^{z_{2}(t-\tau _{22})}-c_{2}(t),\quad t\ne \tau _k,\\ e^{z_1(\tau _k^+)}=(1+h_{1k})e^{z_1(\tau _k)},\\ e^{z_2(\tau _k^+)}=(1+h_{2k})e^{z_2(\tau _k)},\quad k\in {\mathbb {Z}}^+. \end{array} \right. \end{aligned}$$

Suppose that $Z(t)=(z_{1}(t),z_{2}(t))^{T}$ and $Z^{*}(t)=(z_{1}^{*}(t),z_{2}^{*}(t))^{T}$ are any two solutions of system (25). Consider the product system of system (25)

26 $$\begin{aligned} \left\{ \begin{array}{lll} \dot{z}_{1}(t)=\frac{a_{10}(t)}{e^{z_{1}(t-\tau _{10})}+m_{1}(t)} -a_{11}(t)e^{z_{1}(t-\tau _{11})}-a_{12}(t)e^{z_{2}(t-\tau _{12})}-c_{1}(t),\\ \dot{z}_{2}(t)=\frac{a_{20}(t)}{e^{z_{2}(t-\tau _{20})}+m_{2}(t)}-a_{21}(t)e^{z_{1}(t-\tau _{21})} -a_{22}(t)e^{z_{2}(t-\tau _{22})}-c_{2}(t),\\ \dot{z}_{1}^*(t)=\frac{a_{10}(t)}{e^{z_{1}^*(t-\tau _{10})}+m_{1}(t)} -a_{11}(t)e^{z_{1}^*(t-\tau _{11})}-a_{12}(t)e^{z_{2}^*(t-\tau _{12})}-c_{1}(t),\\ \dot{z}_{2}^*(t)=\frac{a_{20}(t)}{e^{z_{2}^*(t-\tau _{20})}+m_{2}(t)}-a_{21}(t)e^{z_{1}^*(t-\tau _{21})} -a_{22}(t)e^{z_{2}^*(t-\tau _{22})}-c_{2}(t),\quad t\ne \tau _k,\\ e^{z_1(\tau _k^+)}=(1+h_{1k})e^{z_1(\tau _k)},\\ e^{z_2(\tau _k^+)}=(1+h_{2k})e^{z_2(\tau _k)},\\ e^{z_1^*(\tau _k^+)}=(1+h_{1k})e^{z_1^*(\tau _k)},\\ e^{z_2^*(\tau _k^+)}=(1+h_{2k})e^{z_2^*(\tau _k)},\quad k\in {\mathbb {Z}}^+. \end{array} \right. \end{aligned}$$

Set $S_1=\{\phi =(z_{1t},z_{2t})^{T}\in C([-\tau ,0],{\mathbb {R}}^{2}):\ln N_i\le z_{it}\le \ln M_i, t\in {\mathbb {R}}^{+}, i=1,2\}$, which is an invariant set of system (26) directly from Remark 7.

Construct a Lyapunov functional $V(t)=V(t,\phi ,\psi )=V\big (t,(z_{1t},z_{2t})^{T},(z_{1t}^{*},z_{2t}^{*})^{T}\big )$ defined on ${\mathbb {R}}^{+}\times S_1\times S_1$ as follows:

$$V(t,\phi ,\psi )= V_{1}(t,\phi ,\psi )+V_{2}(t,\phi ,\psi )+V_{3}(t,\phi ,\psi )+V_{4}(t,\phi ,\psi ),$$

where

$$\begin{aligned} V_{1}(t,\phi ,\psi ) &= \lambda _{1}|z_{1}(t)-z_{1}^{*}(t)|+\lambda _{2}|z_{2}(t)-z_{2}^{*}(t)|,\\ V_{2}(t,\phi ,\psi )&= \lambda _{1}\bigg (\frac{a_{10}^{u}M_{1}}{\big (N_{1}+m_{1}^{l}\big )^{2}}\bigg )^{2} \int _{-2\tau _{10}}^{-\tau _{10}}\int _{t+s}^{t}\big |z_{1}(r)-z_{1}^{*}(r)\big |\,{\mathrm {d}}r\,{\mathrm {d}}s\\&\quad+\,\lambda _{1}\frac{a_{10}^{u}a_{11}^{u}M_{1}^{2}}{\big (N_{1}+m_{1}^{l}\big )^{2}} \int _{-\tau _{11}-\tau _{10}}^{-\tau _{11}}\int _{t+s}^{t}\big |z_{1}(r)-z_{1}^{*}(r)\big |\,{\mathrm {d}}r\,{\mathrm {d}}s\\&\quad+\,\lambda _{1}\frac{a_{10}^{u}a_{11}^{u}M_{1}^{2}}{\big (N_{1}+m_{1}^{l}\big )^{2}}\int _{-\tau _{10}-\tau _{11}}^{-\tau _{10}}\int _{t+s}^{t}\big |z_{1}(r)-z_{1}^{*}(r)\big |\,{\mathrm {d}}r\,{\mathrm {d}}s\\&\quad+\,\lambda _{1}a_{11}^{u2}M_{1}^{2}\int _{-2\tau _{11}}^{-\tau _{11}}\int _{t+s}^{t}\big |z_{1}(r)-z_{1}^{*}(r)\big |\,{\mathrm {d}}r\,{\mathrm {d}}s\\&\quad+\,\lambda _{2}\frac{a_{20}^{u}a_{21}^{u}M_{1}M_{2}}{\big (N_{2}+m_{2}^{l}\big )^{2}}\int _{-\tau _{21}-\tau _{20}}^{-\tau _{21}}\int _{t+s}^{t}\big |z_{1}(r)-z_{1}^{*}(r)\big |\,{\mathrm {d}}r\,{\mathrm {d}}s\\&\quad+\,\lambda _{2}a_{21}^{u}a_{22}^{u}M_{1}M_{2}\int _{-\tau _{21}-\tau _{22}}^{-\tau _{21}}\int _{t+s}^{t}\big |z_{1}(r)-z_{1}^{*}(r)\big |\,{\mathrm {d}}r\,{\mathrm {d}}s,\\ V_{3}(t,\phi ,\psi )&= \lambda _{1}\frac{a_{10}^{u}a_{12}^{u}M_{1}M_{2}}{\big (N_{1}+m_{1}^{l}\big )^{2}} \int _{-\tau _{12}-\tau _{10}}^{-\tau _{12}}\int _{t+s}^{t}\big |z_{2}(r)-z_{2}^{*}(r)\big |\,{\mathrm {d}}r\,{\mathrm {d}}s\\&\quad+\,\lambda _{1}a_{11}^{u}a_{12}^{u}M_{1}M_{2}\int _{-\tau _{12}-\tau _{11}}^{-\tau _{12}}\int _{t+s}^{t}\big |z_{2}(r)-z_{2}^{*}(r)\big |\,{\mathrm {d}}r\,{\mathrm {d}}s\\&\quad+\,\lambda _{2}\bigg (\frac{a_{20}^{u}M_{2}}{\big (N_{2}+m_{2}^{l}\big )^{2}}\bigg )^{2}\int _{-2\tau _{20}}^{-\tau _{20}}\int _{t+s}^{t}\big |z_{2}(r)-z_{2}^{*}(r)\big |\,{\mathrm {d}}r\,{\mathrm {d}}s\\&\quad+\,\lambda _{2}\frac{a_{20}^{u}a_{22}^{u}M_{2}^{2}}{\big (N_{2}+m_{2}^{l}\big )^{2}}\int _{-\tau _{22}-\tau _{20}}^{-\tau _{22}}\int _{t+s}^{t}\big |z_{2}(r)-z_{2}^{*}(r)\big |\,{\mathrm {d}}r\,{\mathrm {d}}s\\&\quad+\,\lambda _{2}\frac{a_{20}^{u}a_{22}^{u}M_{2}^{2}}{\big (N_{2}+m_{2}^{l}\big )^{2}}\int _{-\tau _{20}-\tau _{22}}^{-\tau _{20}}\int _{t+s}^{t}\big |z_{2}(r)-z_{2}^{*}(r)\big |\,{\mathrm {d}}r\,{\mathrm {d}}s\\&\quad+\,\lambda _{2}a_{22}^{u2}M_{2}^{2}\int _{-2\tau _{22}}^{-\tau _{22}}\int _{t+s}^{t}\big |z_{2}(r)-z_{2}^{*}(r)\big |\,{\mathrm {d}}r\,{\mathrm {d}}s,\\ V_{4}(t,\phi ,\psi )&= \lambda _{2}a_{21}^{u}M_{1}\int _{t-\tau _{21}}^{t}\big |z_{1}(s)-z_{1}^{*}(s)\big |\,{\mathrm {d}}s +\lambda _{1}a_{12}^{u}M_{2}\int _{t-\tau _{12}}^{t}\big |z_{2}(s)-z_{2}^{*}(s)\big |\,{\mathrm {d}}s. \end{aligned}$$

By the definitions of $S_1$ and *V*, there is some large enough positive constant *K* such that

$$V(t,\phi ,\psi )\le K.$$

Similar to the argument as that in Zhang et al. (2012), we have

27 $$V(t,\phi ,\psi )\ge \underline{\lambda }|\phi (0)-\psi (0)|_{0},$$

where $$\underline{\lambda }:=\min \{\lambda _{1},\lambda _{2}\}$$,

28 $$V(t,\phi ,\psi )\le \overline{\lambda }\Vert \phi -\psi \Vert ,$$

where

$$\begin{aligned} \overline{\lambda }:&= \lambda _{1}+\lambda _{2}+2\lambda _{1}\tau ^{2}\bigg (\frac{a_{10}^{u}M_{1}}{\big (N_{1}+m_{1}^{l}\big )^{2}}\bigg )^{2} +2\lambda _{1}\tau ^{2}\frac{a_{10}^{u}a_{11}^{u}M_{1}^{2}}{\big (N_{1}+m_{1}^{l}\big )^{2}}\\&\quad+\,2\lambda _{1}\tau ^{2}\frac{a_{10}^{u}a_{11}^{u}M_{1}^{2}}{\big (N_{1}+m_{1}^{l}\big )^{2}}+2\lambda _{1}\tau ^{2}a_{11}^{u2}M_{1}^{2} +2\lambda _{2}\tau ^{2}\frac{a_{20}^{u}a_{21}^{u}M_{1}M_{2}}{\big (N_{2}+m_{2}^{l}\big )^{2}}\\&\quad+\,2\lambda _{2}\tau ^{2}a_{21}^{u}a_{22}^{u}M_{1}M_{2} +2\lambda _{1}\tau ^{2}\frac{a_{10}^{u}a_{12}^{u}M_{1}M_{2}}{\big (N_{1}+m_{1}^{l}\big )^{2}} +2\lambda _{1}\tau ^{2}a_{11}^{u}a_{12}^{u}M_{1}M_{2}\\&\quad+\,2\lambda _{2}\tau ^{2}\bigg (\frac{a_{20}^{u}M_{2}}{\big (N_{2}+m_{2}^{l}\big )^{2}}\bigg )^{2} +2\lambda _{2}\tau ^{2}\frac{a_{20}^{u}a_{22}^{u}M_{2}}{\big (N_{2}+m_{2}^{l}\big )^{2}} +2\lambda _{2}\tau ^{2}\frac{a_{20}^{u}a_{22}^{u}M_{2}^{2}}{\big (N_{2}+m_{2}^{l}\big )^{2}}\\&\quad+\,2\lambda _{2}\tau ^{2}a_{22}^{u2}M_{2}^{2} +\lambda _{2}\tau a_{21}^{u}M_{1} +\lambda _{1}\tau a_{12}^{u}M_{2}, \end{aligned}$$

and for $\forall \,\bar{\phi }=(\bar{z}_{1t},\bar{z}_{2t})^{T}, \bar{\psi }=(\bar{z}_{1t}^{*},\bar{z}_{2t}^{*})^{T}, \hat{\phi }=(\hat{z}_{1t},\hat{z}_{2t})^{T}, \hat{\psi }=(\hat{z}_{1t}^{*},\hat{z}_{2t}^{*})^{T}\in S_1$, it follows that

$$\big |V(t,\bar{\phi },\bar{\psi })-V(t,\hat{\phi },\hat{\psi })\big | \le \overline{\lambda }(\Vert \bar{\phi }-\hat{\phi }\Vert +\Vert \bar{\psi }-\hat{\psi }\Vert ).$$

So condition **(2)** in Lemma 2 is satisfied. In view of (27)–(28), let $u, v\in C({\mathbb {R}}^{+},\,{\mathbb {R}}^{+})$, $u(s)=\underline{\lambda }s$, $v(s)=\overline{\lambda }s$, thus condition **(1)**$'$ in Remark 2 is satisfied.

From article Zhang et al. (2012), for $t\ne \tau _k$, $k\in {\mathbb {Z}}^+$, calculating the upper right derivative of *V* along the solution of system (26), we have

29 $$D^{+}V(t,\phi ,\psi )\le -\gamma V(t,\phi ,\psi ),$$

where $$\gamma :=\frac{\chi \underline{\lambda }|\phi (0)-\psi (0)|_0}{K}$$, $$\chi :=\min \{\frac{\Theta }{\lambda _1},\frac{\Theta }{\lambda _2}\}$$ and $$\Theta :=\min \{\lambda _{1}\alpha _{1}-\lambda _{2}\beta _{1},\lambda _{2}\alpha _{2}-\lambda _{1}\beta _{2}\}$$.

For $t=\tau _k$, $k\in {\mathbb {Z}}^+$, we have

30 $$\begin{aligned} V(\tau _k^+,\phi ,\psi )&= V_{1}(\tau _k^+,\phi ,\psi )+V_{2}(\tau _k^+,\phi ,\psi )+V_{3}(\tau _k^+,\phi ,\psi )+V_{4}(\tau _k^+,\phi ,\psi ) \\&= V_{1}(\tau _k^+,\phi ,\psi )+V_{2}(\tau _k,\phi ,\psi )+V_{3}(\tau _k,\phi ,\psi )+V_{4}(\tau _k,\phi ,\psi ) \\&= \sum _{i=1}^2\lambda _{i}|z_{i}(\tau _k^+)-z_{i}^{*}(\tau _k^+)|+V_{2}(\tau _k,\phi ,\psi )+V_{3}(\tau _k,\phi ,\psi )+V_{4}(\tau _k,\phi ,\psi ) \\&= \sum _{i=1}^2\lambda _{i}|z_{i}(\tau _k)-z_{i}^{*}(\tau _k)|+V_{2}(\tau _k,\phi ,\psi )+V_{3}(\tau _k,\phi ,\psi )+V_{4}(\tau _k,\phi ,\psi ) \\&= V_{1}(\tau _k,\phi ,\psi )+V_{2}(\tau _k,\phi ,\psi )+V_{3}(\tau _k,\phi ,\psi )+V_{4}(\tau _k,\phi ,\psi ) \\&= V(\tau _k,\phi ,\psi ). \end{aligned}$$

In view of (29)–(30), condition **(3)** in Lemma 2 is satisfied.

By Lemma 2, system (2) admits a unique uniformly asymptotically stable positive almost periodic solution $(z_{1}(t),z_{2}(t))^{T}$. This completes the proof. $\square$

### *Remark 8*

Without (*F*), system (2) also admits a unique uniformly asymptotically stable positive almost periodic solution. So Theorem  2 extends the corresponding result in Zhang et al. (2012). Further, Theorem 2 gives the sufficient conditions for the uniform asymptotical stability of a unique positive almost periodic solution of system (2). Therefore, Theorem 2 provides a possible method to study the existence, uniqueness and stability of positive almost periodic solution of the models with impulsive perturbations and pure-delays in biological populations.

### *Remark 9*

In the last two decades, the method of constructing a Lyapunov functional has been extensively used in the study of stability of the deterministic models (Xue et al. 2015; Tian et al. 2015; Zhang et al. 2015; Liu and Xu 2004; Liu et al. 2006; Li and Yang 2009; Gan and Lin 2012; Wu et al. 2009; Zhang et al. 2012). However, there have been numerous relevant works using the Lyapunov functional method in stochastic systems, see Shang (2014, 2015, 2016). The methods used in this paper can be extended to study the permanence and existence of a unique uniformly asymptotically stable positive almost periodic solution of the stochastic models with impulsive perturbations in biological populations.

## An example and numerical simulations

### *Example 1*

Consider the following Schoener’s competition model with pure-delays and impulsive effects:

$$\begin{aligned} \left\{ \begin{array}{ll} \dot{x}_{1}(t)=x_{1}(t)\bigg \{\frac{1}{x_{1}(t-0.0001)+2}-a_{11}(t)x_{1}(t-0.0001)-a_{12}(t)x_{2}(t-0.0002)-c_{1}(t)\bigg \},\\ \dot{x}_{2}(t)=x_{2}(t)\bigg \{\frac{1}{x_{2}(t-0.0001)+2}-a_{21}(t)x_{1}(t-0.0002)-a_{22}(t)x_{2}(t-0.0001)-c_{2}(t)\bigg \},\quad t\ne \tau _{k},\\ \Delta x_{1}(\tau _{k})=0.5x_{1}(\tau _{k}),\\ \Delta x_{2}(\tau _{k})=0.4x_{2}(\tau _{k}),\quad \{\tau _{k}:k\in {\mathbb {Z}}\}\subset \{5k:k\in {\mathbb {Z}}\}, \end{array} \right. \end{aligned}$$

where $a_{11}(t)=a_{22}(t)=0.35+0.05\cos (\sqrt{3}t)$, $a_{12}(t)=a_{21}(t)\equiv 0.0001$, $c_{1}(t)=0.30005+0.00005\sin (\sqrt{2}t)$, $c_{2}(t)=0.30005+0.00005\cos (\sqrt{2}t)$, $t\in {\mathbb {R}}$. Then the above system is permanent and has a unique uniformly asymptotically stable almost periodic solution.

### *Proof*

**Fig. 1:**
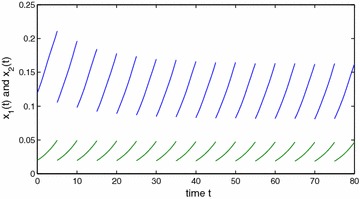
State variables $x_1$ and $x_2$ of Example 1. *Blue line* $x_1(s)=0.12$, $s\in [-1,0]$; *Green line* $x_2(s)=0.02$, $s\in [-1,0]$

**Fig. 2:**
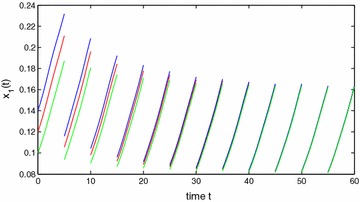
Stability of state variable $x_1$ of Example 1. *Blue line*  $x_1(s)=0.14$, $s\in [-1,0]$; *Red line* $x_1(s)=0.12$, $s\in [-1,0]$; *Green line* $x_1(s)=0.10$, $s\in [-1,0]$

**Fig. 3:**
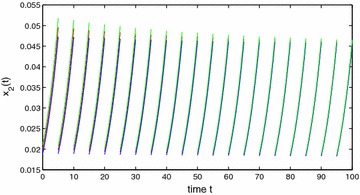
Stability of state variable $x_2$ of Example 1. *Blue line* $x_2(s)=0.018$, $s\in [-1,0]$; *Red line* $x_2(s)=0.002$, $s\in [-1,0]$; *Green line* $x_1(s)=0.022$, $s\in [-1,0]$

Obviously, $a_{10}^{u}=a_{20}^{u}=1$, $a_{10}^{l}=a_{20}^{l}=1$, $a_{11}^{u}=a_{22}^{u}=0.4$, $a_{11}^{l}=a_{22}^{l}=0.3$, $a_{12}^{u}=a_{21}^{u}=0.0001$, $a_{12}^{l}=a_{21}^{l}=0.0001$, $c_{1}^{u}=c_{2}^{u}=0.3001$, $c_{1}^{l}=c_{2}^{l}=0.3$, $\theta =2$, $\eta _1\approx 0.4$, $\eta _2=4$, $\xi _1\approx 0.3$, $\xi _2\approx 0.7$, $A=1$. By calculation, we obtain that $y_{1}^{*}\approx 0.7256$, $y_{2}^{*}\approx 0.5421$, $\min \{r_{1}^{l},r_{2}^{l}\}\ge 0.03>0$, $y_{1*}=y_{2*}\ge 0.01$. So $(H_1)$ holds. Further, we also get that

$$\alpha _{1}\ge 0.03,\quad \alpha _{2}\ge 0.02,\quad \max \{\beta _{1},\beta _{2}\}\le 0.00007,$$

which implies that $(H_2)$ is satisfied for $\lambda _{1}=\lambda _{2}=1$. It is easy to verify that $(H_1)$–$(H_2)$ are satisfied and the result follows from Theorems  1 and 2 (see Figs. 1, 2, 3). This completes the proof. $\square$

### *Remark 10*

In Example 1, the impulse coefficients ($h_{1k}=0.5$ and $h_{2k}=0.6$) do not satisfy (*F*). So it is impossible to obtain the permanence and existence of a unique uniformly asymptotically stable positive almost periodic solution of system (1) by the result in Zhang et al. (2012). Therefore, the work in this paper improves the results in paper Zhang et al. (2012).

## Conclusion

By using the comparison theorem and the Lyapunov method of the impulsive differential equations, sufficient conditions are obtained for the permanence and existence of a unique uniformly asymptotically stable positive almost periodic solution in a class of impulsive Schoener’s competition model with pure-delays. Proposition 2 and Theorem 2 imply that the values of impulse coefficients $h_{ik}\,(i=1,2,3)$ and the number of the impulse points $\tau _k$ in each interval of length 1 are harm for the permanence and existence of a unique uniformly asymptotically stable positive almost periodic solution for a class of impulsive Schoener’s competition model with pure-delays. The main results obtained in this paper are completely new and the method used in this paper provides a possible method to study the permanence and existence of a unique uniformly asymptotically stable positive almost periodic solution of the models with impulsive perturbations and pure-delays in biological populations.
